# *In vivo* characterization of brain tumor biomechanics: magnetic resonance elastography in intracranial B16 melanoma and GL261 glioma mouse models

**DOI:** 10.3389/fonc.2024.1402578

**Published:** 2024-09-11

**Authors:** Anastasia Janas, Jakob Jordan, Gergely Bertalan, Tom Meyer, Jan Bukatz, Ingolf Sack, Carolin Senger, Melina Nieminen-Kelhä, Susan Brandenburg, Irina Kremenskaia, Kiril Krantchev, Sanaria Al-Rubaiey, Susanne Mueller, Stefan Paul Koch, Philipp Boehm-Sturm, Rolf Reiter, Daniel Zips, Peter Vajkoczy, Gueliz Acker

**Affiliations:** ^1^ Department of Neurosurgery, Charité – Universitätsmedizin Berlin, Corporate Member of Freie Universität Berlin and Humboldt Universität zu Berlin, Berlin, Germany; ^2^ Berlin Institute of Health at Charité – Universitätsmedizin Berlin, Berlin, Germany; ^3^ Department of Radiation Oncology and Radiotherapy, Charité – Universitätsmedizin Berlin, Corporate Member of Freie Universität Berlin and Humboldt Universität zu Berlin, Berlin, Germany; ^4^ Department of Radiology, Charité – Universitätsmedizin Berlin, Corporate Member of Freie Universität Berlin and Humboldt Universität zu Berlin, Berlin, Germany; ^5^ Department of Neurology and Experimental Neurology, Charité – Universitätsmedizin Berlin, Corporate Member of Freie Universität Berlin and Humboldt Universität zu Berlin, Berlin, Germany; ^6^ Charité 3R – Replace | Reduce | Refine, Charité – Universitätsmedizin Berlin, Corporate Member of Freie Universität Berlin and Humboldt Universität zu Berlin, Berlin, Germany; ^7^ NeuroCure Cluster of Excellence and Charité Core Facility 7T Experimental MRIs, Charité – Universitätsmedizin Berlin, Corporate Member of Freie Universität Berlin and Humboldt Universität zu Berlin, Berlin, Germany

**Keywords:** elastography, brain tumor, B16 melanoma, brain metastases, glioma, biomechanical properties

## Abstract

**Introduction:**

Magnetic Resonance Elastography (MRE) allows the non-invasive quantification of tumor biomechanical properties *in vivo*. With increasing incidence of brain metastases, there is a notable absence of appropriate preclinical models to investigate their biomechanical characteristics. Therefore, the purpose of this work was to assess the biomechanical characteristics of B16 melanoma brain metastases (MBM) and compare it to murine GL261 glioblastoma (GBM) model using multifrequency MRE with tomoelastography post processing.

**Methods:**

Intracranial B16 MBM (n = 6) and GL261 GBM (n = 7) mouse models were used. Magnetic Resonance Imaging (MRI) was performed at set intervals after tumor implantation: 5, 7, 12, 14 days for MBM and 13 and 22 days for GBM. The investigations were performed using a 7T preclinical MRI with 20 mm head coil. The protocol consisted of single-shot spin echo-planar multifrequency MRE with tomoelastography post processing, contrast-enhanced T1- and T2-weighted imaging and diffusion-weighted imaging (DWI) with quantification of apparent diffusion coefficient of water (ADC). Elastography quantified shear wave speed (SWS), magnitude of complex MR signal (T2/T2*) and loss angle (φ). Immunohistological investigations were performed to assess vascularization, blood-brain-barrier integrity and extent of glucosaminoglucan coverage.

**Results:**

Volumetric analyses displayed rapid growth of both tumor entities and softer tissue properties than healthy brain (healthy: 5.17 ± 0.48, MBM: 3.83 ± 0.55, GBM: 3.7 ± 0.23, [m/s]). SWS of MBM remained unchanged throughout tumor progression with decreased T2/T2* intensity and increased ADC on days 12 and 14 (p<0.0001 for both). Conversely, GBM presented reduced φ values on day 22 (p=0.0237), with no significant alterations in ADC. Histological analysis revealed substantial vascularization and elevated glycosaminoglycan content in both tumor types compared to healthy contralateral brain.

**Discussion:**

Our results indicate that while both, MBM and GBM, exhibited softer properties compared to healthy brain, imaging and histological analysis revealed different underlying microstructural causes: hemorrhages in MBM and increased vascularization and glycosaminoglycan content in GBM, further corroborated by DWI and T2/T2* contrast. These findings underscore the complementary nature of MRE and its potential to enhance our understanding of tumor characteristics when used alongside established techniques. This comprehensive approach could lead to improved clinical outcomes and a deeper understanding of brain tumor pathophysiology.

## 1 Introduction

Malignant brain tumors, including primary brain tumor types like glioblastoma (GBM) or brain metastases (BM), represent a significant global health challenge due to limited treatment options (1, 2). GBM, a WHO Grade 4 malignancy, accounts for 45.2% of primary malignant brain and central nervous system (CNS) tumors, being the predominant primary brain tumor in adults (3, 4). Despite considerable research efforts, the average survival time for patients diagnosed with GBM remains a dismal 12 to 15 months. The prognosis for patients with BM is similarly bleak. It is noteworthy that the incidence of BMs in patients with solid tumors is estimated at 20-30%, with rising tendency due to advancements in systemic treatment strategies (5, 6). Melanoma brain metastases (MBM) are of particular concern. Melanoma ranks as the third most frequent cancer to spread to the brain, and MBMs are characterized by fast, aggressive growth, resulting in a median survival of a mere 4 months (7). Notwithstanding recent scientific advances, comprehensive understanding of these brain tumors remains elusive. Animal models contribute to the understanding of the disease progression, but preclinical models that accurately replicate intracranial melanoma metastases are rare. The models of metastases published herein involve injecting tumor cells into the left cardiac ventricle (8–11). While this approach allows for a more realistic formation of brain metastases, the unpredictable nature of the model poses challenges for certain experimental setups, particularly those assessing biomechanical properties. Different brain areas exhibit distinct cellular compositions, resulting in varied biomechanical tumor properties (12). Therefore, models utilizing stereotactic injection in the brain are more favorable for such studies. Although well-established intracranial mouse models exist for GBM, a robust model for intracranial MBM has yet to be developed. This underscores the need for further experimental research in this area.

The biomechanical properties of tumor tissue, relative to healthy brain tissue, are crucial in understanding intracranial tumor progression. Recent studies have emphasized the importance of brain mechanical properties for understanding cancer development in neural tissue (12). Biomechanical brain tumor hallmarks include loss of tensional homeostasis, resulting in altered tissue properties driven by changes in tumor cell morphology and the composition of the microenvironment (13). Historically, malignant brain tumors were thought to be associated with increased tumor stiffness compared to healthy brain tissue (14, 15). In the last decade, however, it was shown that certain brain tumors (such as GBM), present softer tissue characteristics compared to healthy brain parenchyma (16, 17). Reiss-Zimmermann et al. provided initial insights into the biomechanical properties of various brain metastases (BM) in a clinical context (18). However, among the brain metastases assessed, only one was a melanoma brain metastasis. This limited representation does not allow for clear conclusions about the specific biomechanical properties of MBM. Consequently, little is known about the *in vivo* biomechanical properties of MBM, warranting further research in the area.

From a translational perspective, it’s imperative to employ techniques capable of non-invasively assessing tissue characteristics. At present, magnetic resonance elastography (MRE) is the preferred modality to assess biomechanical properties of brain tissue *in vivo* and non-invasively (19). In MRE, mechanical waves are generated in the tissue of interest using external mechanical vibration. The induced displacement is then encoded using a conventional MRI scanner and the viscoelasticity properties of the tissue are calculated by inverting the measured wave field (20).

MRE has already proven valuable in understanding the biomechanics of brain tumors, both in human patients and animal models (21–23). In preclinical research, several groups already used MRE to assess longitudinal stiffness changes primarily in GBM-bearing mouse models (17, 21, 22, 24, 25). Schregel et al. demonstrated that glioma is softer than healthy brain tissue highlighting the direct relationship between tumor softness and tumor progression (21). However, preclinical studies addressing the biomechanical characterization of MBM are limited, and as noted earlier, resulting in biophysical properties of MBM being largely unexplored.

A significant setback in preclinical MRE research is the lack of time efficient scanning protocols for rodent brains. In clinical settings, cerebral MRE can be completed in about 2 minutes for a full three-dimensional (3D) wave field measurement at a single drive frequency (26). In contrast, MRE for mouse brains can exceed 30 minutes for the same process (26). This time disparity makes conducting longitudinal studies with large numbers of animals challenging. Additionally, many animal studies compromise on image resolution and use a limited range of drive frequencies to keep the total scan time per animal reasonable (27). To address these issues, we’ve recently introduced cerebral MRE in the mouse based on fast, single-shot echo-planar-imaging (EPI) and tomoelastography post processing (26). EPI-MRE in the mouse can measure the 3D displacement field for a single drive frequency within 30 seconds resulting in significantly reduced measurement time. This significant reduction in scan time is especially advantageous for longitudinal brain tumor studies involving many animals.

Our central hypothesis suggests that the biomechanical properties of MBM and GBM are distinct, potentially serving as a differential factor in mouse models. Our hypothesis is founded on empirical observations of their properties during surgical resection (i.e. direct biomechanical assessment through palpation). GBM, typically softer and more infiltrative, presents a heterogeneous texture marked by necrosis, hemorrhage, and variable cell densities, complicating differentiation from healthy brain tissue upon resection (28). In contrast, MBM usually appears firmer and more defined, though its consistency can vary due to internal factors like hemorrhage or necrosis (29). These biomechanical differences, observable through *in vivo* viscoelastic measurements, could significantly advance our understanding of tumor characteristics and progression. Understanding these differences could lay the groundwork for future research, particularly in assessing tumor response to treatments such as stereotactic radiosurgery.

In this study, we systematically analyze the *in vivo* viscoelastic properties of MBM and compare them with those of the well-established GBM mouse model. Our overarching objective is to introduce MRE with tomoelastography post processing for brain tumor characterization. By employing this advanced imaging technique, we aim to elucidate the dynamics of MBM biomechanical alterations through a longitudinal study design.

## 2 Materials and methods

### 2.1 Cells

Murine B16F0 cells for the MBM (Sigma-Aldrich, Darmstadt, Germany) and murine GL261 cells for the GBM model (Leibnitz-Institut DSMZ) were used. Cells were cultivated in Dulbecco’s Modified Eagle Medium (DMEM; [+] 4.5g/L D-Glucose, L-Glutamine; [+] Pyruvate; Gibco^®^) supplemented with 10% fetal bovine serum (FBS) and 1% Penicillin/Streptomycin in a humidified atmosphere of CO_2_/air (5%/95%) at 37°C. B16 cells were cultivated for 48 hours until 70% confluence and GL261 cells were cultivated for 60 hours until 70% confluence was reached. The number of viable cells was quantified using a hemocytometer counter (Neubauer) under a light microscope with 10× magnification. Afterwards, cells were diluted in PBS to the aimed density of 300 cells/µl (B16F0) and 20,000 cells/μl (GL261) kept on ice until implantation.

### 2.2 Animal models

Thirteen female C57BL/6 mice (10 ± 2 weeks old, 20 ± 2 g, Charles River Laboratories, Sulzfeld, Germany; GBM n = 7, MBM n = 6) were used. Animals were maintained in animal care facilities in a temperature-regulated room with a 12h-light-dark cycle and supplied with water and standard mouse chow ad libitum. The experiments were performed according to German Laws for Animal Protection and controlled by LaGeSo (Berlin, Germany) under the registration number G0130/20. Arrive 2.0 guidelines were followed.

Tumor cell implantation was performed as previously described (30). In short, anesthetized animals were positioned in a fixation frame and the skull was exposed. Using a 1 μL Hamilton syringe tumor cells were administered into the right striatum (2 mm lateral, 1 mm anterior, and 3 mm deep from the bregma) over 15 min.

### 2.3 MRI

**Figure 1:**
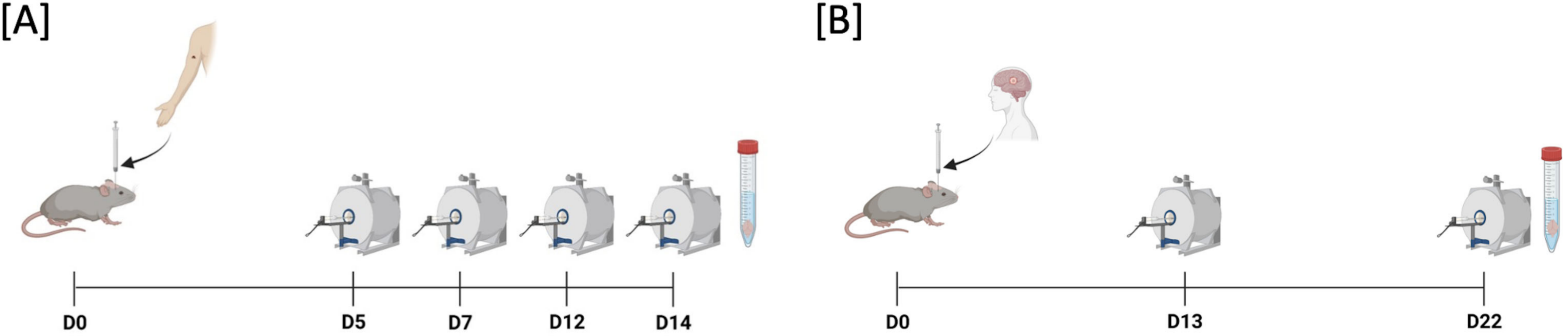
Experimental setup. **(A)** B16 melanoma cells (n = 300) were implanted into the right striatum of C57BL/6 mice via stereotactic implantation (d0). MRI investigations were performed on days 5, 7, 12, and 14 using a 7T preclinical scanner. The applied imaging protocol included magnetic resonance elastography (MRE) with tomoelastography post processing, contrast-enhanced T1-weighted magnetic resonance imaging (MRI), T2-weighted MRI, and diffusion-weighted MRI measuring the apparent diffusion coefficient (ADC). **(B)** C57BL/6 mice received 20,000 GL261 cells via stereotactic implantation (d0). MRI investigations were performed on days 13 and 22 after implantation following same MRI protocol as in B16 melanoma mouse model.

Imaging was performed on a 7T preclinical MR scanner (BioSpec 70/20 USR, Bruker, Germany and Paravision 6.0.1 software) using a 20 mm head coil (RAPID Biomedical GmbH, Rimpar, Germany). Animals were measured either on days 5, 7, 12, and 14 after MBM implantation (n = 6) or on days 13 and 22 after GBM implantation (n = 7; **Figures 1A, B**). The varying timepoints are attributed to the faster growth rate of the B16 tumor, which reaches a tumor volume by day 14 that is comparable to the volume of GL261 by day 22. Mice were anesthetized with isoflurane (1.0–1.5%, CP Pharma, Burgdorf, Germany) diluted in 30% O_2_ and 70% N_2_O. The applied protocol consisted of (1) T1-weighted imaging with gadolinium contrast agent (200µl, intraperitoneal application; Gadovist^®^ for MBM and Magnevist^®^ for GBM, Bayer AG, Leverkusen, Germany), (2) T2-weighted imaging, (3) diffusion-weighted imaging (DWI) with reconstruction of apparent diffusion coefficient (ADC) and (4) MRE with tomoelastography post processing. Acquisition parameters are summarized in **Table 1**. Tumor volume was measured by manual segmentation on contrast-enhanced T1 images using Analyze 10.0 (AnalyzeDirect, Inc., Overland, Park, KS, USA). The T2-weighted sequence was utilized to assess the tumor volume and the peritumoral edema. For calculating the edema, the difference in volume between T2 and contrast-enhanced T1 images was expressed as a percentage of the total T1 volume, as described previously (30).

**Table 1:** Overview of acquisition parameters of applied MRI protocol.

Acquisition parameters	Sequence		
	T1 + CA	T2	DWI + ADC
**TR (ms)**	1000	4200	3000
**TE (ms)**	10	36	28
**FoV (mm^2^)**	19.2 x 19.2	19.2 x 19.2	19.2 x 19.2
**In plane resolution (mm^2^)**	0.1 x 0.1	0.1 x 0.1	0.15 x 0.1
**Slice thickness (mm)**	0.5	0.5	1
**Averages**	3	3	4
**B values (s/mm^2^)**			0, 650, 1300
**Diffusion directions**			3 per b value 3 images per direction

CA, Contrast Agent; DWI, diffusion weighted imaging; ADC, apparent diffusion coefficient of water; TR, repetition time; TE, echo time; FoV, field of view.

### 2.4 MRE

The used MRE technique is described in detail in (26). Mechanical vibration was generated by a custom-made driver system using a nonmagnetic piezoceramic actuator (CEDRAT Technologies, Meylan Cedex, France). Vibrations of 1000, 1200, and 1400 Hz with a peak-to-peak displacement of 60 µm were transferred to the head holder via a carbon fiber. 3D wave fields were recorded using a modified single-shot spin echo-planar imaging sequence incorporating a sinusoidal motion-encoding gradient (MEG) with the ‘‘SLIM” acquisition strategy (31). Eight time points, equally spaced over a full oscillation period, were measured to assess the wave field dynamics. Further acquisition parameters were repetition time (TR) = 3000 ms, echo time (TE) = 50 ms, field of view (FoV) = 16 × 16 mm^2^, 0.2 × 0.2 mm^2^ in-plane resolution, 0.5 mm slice thickness, and 3 averages. The total acquisition time per measurement with 11 consecutive axial slices, 3 wave field components, 8 wave dynamics, and 3 averages was 4 min. Each measurement was repeated 3 times and averaged with each other to further increase image quality and the stability of the obtained stiffness values. Thus, the total measurement time with 3 × 3 averages was 12 min per animal. Data post processing was based on the tomoelastography pipeline (32), which is publicly available (33). In brief, MRI images were phase unwrapped and Fourier transformed along each MEG direction and across the wave duration, selecting the fundamental frequency. The resulting wavefields were inverted and then averaged using multifrequency dual elasto-visco (MDEV) inversion (27), generating maps of the loss angle of the complex shear modulus (φ in radian), as well as the wave- number (k)MDEV inversion (34), generating maps of the shear wave speed (SWS in m/s), respectively. For consistency with the literature, we will use the term stiffness only for discussion of relative differences in SWS, the actually measured quantity in MRE with tomoelastography post processing, and use SWS otherwise (19). The loss angle φ can be used as a marker of viscosity that is interpreted as indication of solid-rigid properties or viscous-fluid properties for lower and higher values, respectively (35). Additionally, the MRE magnitude signal provided a T2/T2* weighted (w) contrast used as a proxy to assess relative differences in water content as described in (35). Importantly, T2/T2*w image contrast varies depending on the imaging system, protocol parameters, and scanner hardware, making it unsuitable for providing absolute values that can be directly compared across different MRI systems. However, within the same system and with consistent sequence protocols, relative changes in these values are meaningful as they reflect changes in the tissue water pool and the mobility of water molecules. In this context, we focused on reporting the percentage of relative changes observed in the longitudinal assessment of the tumor entities, using p-values to demonstrate the significance of the changes. Regions of interest (ROI) enclosing the whole tumor were defined based on T2/T2*w images and applied to the corresponding SWS and φ maps. Mirrored ROIs were placed in the healthy tissue of the contralateral hemisphere. The biomechanical characteristics of the tumor region on days 13 and 22 after GBM implantation (n = 7) and on days 5, 7, 12, and 14 days after MBM implantation (n = 6) were compared to those in healthy brain tissue on the contralateral hemisphere.

### 2.5 Immunohistochemistry

Anaesthetized animals (GBM n = 7; d22 after implantation, MBM n = 5 (one animal was excluded due to unsatisfactory perfusion quality); d14 after implantation) were perfused intracardially with 4% paraformaldehyde (PFA). Whole brains were harvested, post-fixed in 4% PFA for 24 hours, and dehydrated in sucrose solutions with rising concentrations. Brains were frozen in liquid nitrogen and embedded in 2% gelatine to obtain coronal sections with 10 μm thickness using a cryostat (Microm HM 505 E; Microm, Walldorf, Germany).

To assess the tumor vasculature and blood brain barrier (BBB) integrity the following stainings were performed: CD31 for tumor vasculature, desmin for the pericyte coverage and albumin as surrogate for vessel leakiness. Prior to the staining autoimmunofluorescense blocker (CAT #2160, Millipore, Darmstadt, Germany) was used according to the manufacturer’s instructions. Sections (n = 21 for GBM; n = 15 for MBM) were fixed in methanol at -20°C for 10 min and blocked in 1% Casein for 30 min. Primary antibodies were diluted in Casein 0.5% and incubated for 2 hours in a dark humid chamber: rat anti-CD31 (1:50; CAT #550274, BD Pharmingen, Heidelberg, Germany), rabbit anti-Desmin (1:100; CAT #ab15200, Abcam), goat anti-albumin (1:100; CAT #NB600-41532, Abcam). Sections were washed in 0.5% Casein (3 × 5 min) and corresponding secondary antibodies were applied: anti-rat Cy3 (CAT #712-165-153, Dianova), anti-rabbit FITC (CAT #711-545-152, Dianova) and anti-goat Cy5 (CAT #705-605-147, Dianova), [1:200 in 0.5% Casein]. Tissue samples were incubated for 1.5 hours at room temperature and washed with PBS and Millipore water for 3 × 5 min, respectively. Finally, DAPI-containing mounting medium (Dianova) was applied on the slides.

Quantitative analysis of glycosaminoglycan (GAG) content in tumor and brain tissue was conducted using the Alcian Blue/Periodic Acid-Schiff (PAS) Stain Kit (CAT #ab245876, Abcam), adhering to the manufacturer’s protocol. GAGs are known for their hydrophilic nature thus being crucial to the structure of the brain’s extracellular matrix. Consequently, the extent of GAG coverage serves as a quantifiable surrogate marker for GAG-bound water content within the tumor stroma.

To ensure a thorough examination of the tumor area, images were captured from a minimum of three unique sections for every staining type, leading to a collection of 12-15 representative images for each animal. These images were taken using a Zeiss Axio Observer Z1 immunofluorescence microscope (Zeiss MicroImaging GmbH) at 20× magnification. Subsequently, ImageJ Software (U. S. National Institutes of Health, Bethesda, Maryland, USA) was utilized for analysis.

For the analysis of tumor vasculature, several parameters were assessed. Vessel density was calculated as the total number of vessels divided by the analyzed tumor area. Average vessel size was determined by dividing the total vessel area by the number of vessels, while vessel area fraction was derived as the percentage of the total vessel area relative to the total tumor area. Furthermore, the integrity of the BBB was assessed through pericyte coverage by determining the percentage of vessels demonstrating desmin positivity relative to the total number of vessels. Additionally, albumin permeability was assessed by calculating the percentage of vessels that were albumin-positive relative to the total vessel count, or by comparing the area occupied by albumin to the total tumor volume.

### 2.6 Statistical analysis

Results are presented as mean ± standard deviation. A one-way ANOVA with Bonferroni correction was used to identify differences in biomechanical properties among tumor and healthy brain regions. Paired Student’s *t*-tests were performed to test for differences between tumor volumes on different time points. Comparison of histological characteristics between the tumor entities was performed using unpaired Student’s t-test. P values less than 0.05 were considered significant. All analyses were performed using GraphPad Prism (v 10.0 GraphPad Software, San Diego, CA, USA). Correlations were tested using RStudio’s Hmisc library and rcorr function.

## 3 Results

### 3.1 Histological appraisal of tumor models revealed tumor specific characteristics

**Figure 2:**
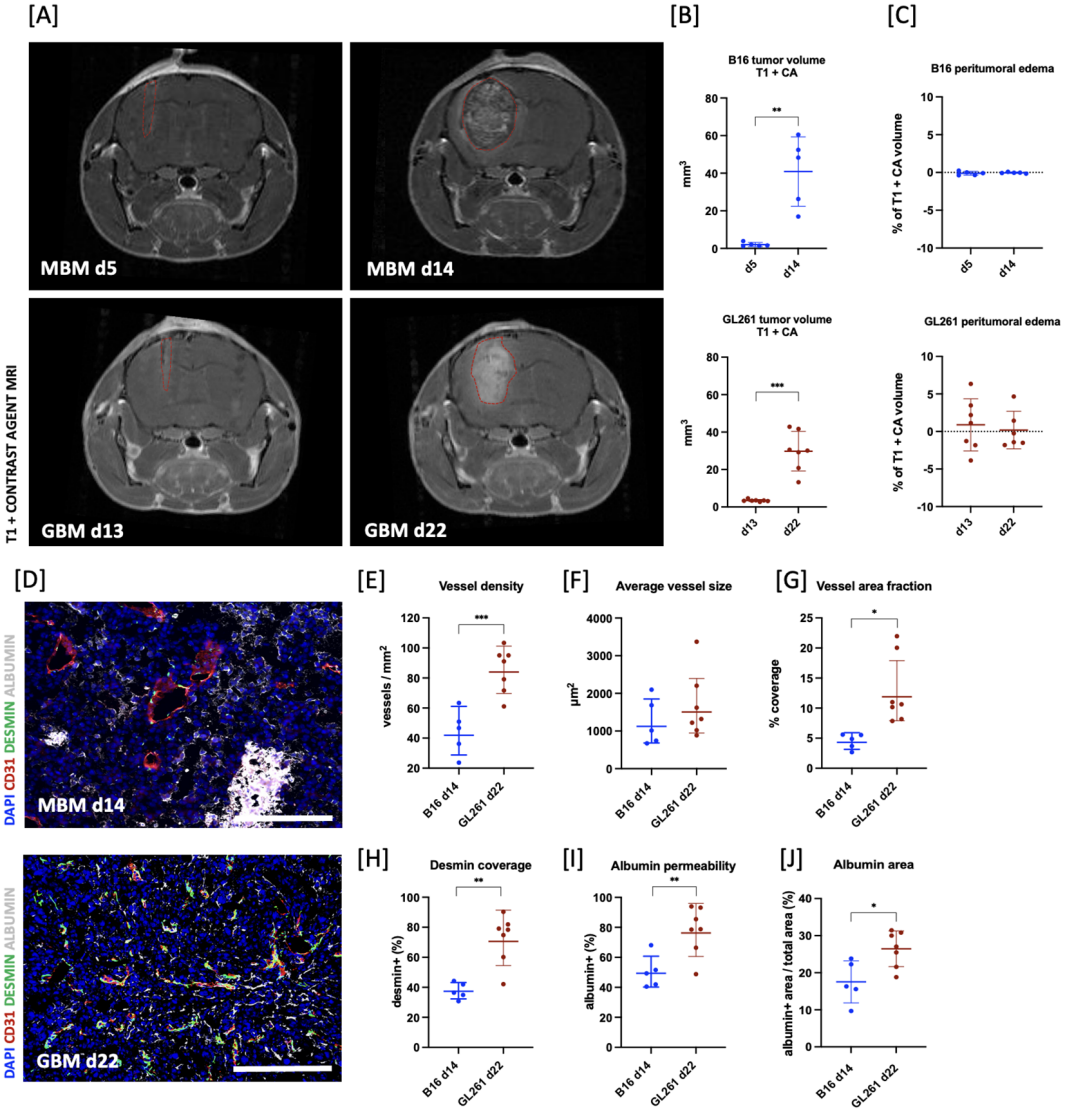
Validation and comparison of tumor models. **(A)** Coronal T1-weighted MRI images of two representative animals on days 5 and 14 after melanoma brain metastases (MBM; B16 in graphs) or 13 and 22 after glioblastoma (GBM; GL261 in graphs) implantation, highlighting tumor type characteristic rapid growth dynamic. **(B)** Volumetric analysis of tumor volumes using contrast-enhanced T1 sequence (T1 + CA) on days 5 and 14 for MBM (n = 5) and days 13 and 22 for GBM (n = 7) revealed a significant increase in tumor volume within the observed timeframe (MBM:**p = 0.0015; GBM: ***p = 0.0005). **(C)** Quantification of edema size showed no significant changes in either tumor entity; however, a large variability within the GBM group was observed. Edema was calculated as the difference in the tumor volume between the T2- and T1-weighted MRI images and presented as the percentage of total tumor volume. **(D)** Representative immunohistological images illustrating disturbed structural integrity of the tumor vasculature (DAPI: blue, CD31: red, DESMIN: green, ALBUMIN: white; scale bar = 200 µm) of MBM on day 14 (upper) or GBM on day 22 (lower) after implantation. **(E–J)** Histological appraisal revealed higher vessel density in GBM compared to the vasculature of MBM (***p = 0.0009). Simultaneously, whilst the fraction of tumor area covered with vessels, shown as coverage (%), was lower in MBM (*p = 0.01), average vessel size was comparable for both tumor entities. MBM vessels had significantly lower pericyte coverage (**p = 0.001), but GBM vessels presented with higher leakiness for albumin (**p = 0.0077 and *p = 0.0149). All histological analyses were performed on n = 7 animals for GBM and n = 5 animals for MBM.

The tumor models used in this study were histologically assessed regarding their representativeness. In line with previous reports (36, 37), the B16 MBM tumor model presented a significant increase in tumor volume between days 5 and 14 (day 5: 2.0 ± 1.1 mm^3^ vs. day 14: 40 ± 18 mm^3^, **p = 0.0015; **Figures 2A, B**). No peritumoral edema was evident throughout tumor development (d5: -0.11 ± 0.25% vs. d14: -0.048 ± 0.09% of tumor volume, p = 0.3827; **Figure 2C**), whilst tumor growth was accompanied by tumor type characteristic intratumoral and peritumoral hemorrhages. Analysis of the tumor vasculature revealed multiple large vessels together with microvessels and an overall low vessel density (44 ± 15 vessels per mm^2^), with blood vessels contributing 4.4 ± 1.3% to the tumor area. The average vessel sizes ranged from 672 µm^2^ to 2097 µm^2^ with a mean of 1240 ± 620 µm^2^ (**Figures 2D–G**). Pericyte coverage analysis indicated that 37.7 ± 5.4% of vessels were associated with pericytes. Moreover, approximately 50 ± 11% of vessels exhibited positive albumin staining. The analysis further showed that albumin covered 17.0 ± 5.7% of the total area (**Figures 2H–J**).

GBM was observed to progress rapidly between days 13 and 22 after implantation (day 13: 3.45 ± 0.22 mm^3^ vs. day 22: 29.8 ± 3.4 mm^3^, ***p = 0.0005; **Figures 2A, B**). Quantification of peritumoral edema showed no significant changes, however displayed a large variability (day 13: 0.89 ± 3.5% vs. day 22: 0.19 ± 2.5% of tumor volume; p = 0.4253; **Figure 2C**). The tumor vasculature exhibited high vessel density (85.2 ± 5.7 vessels per mm^2^) and a large vessel area (12.8 ± 2.2% of the tumor area), with varying average vessel sizes ranging from 880 µm^2^ to 3400 µm^2^ and a mean of 1663 µm^2^ ± 870 µm^2^ (**Figures 2D–G**). Assessment of pericyte coverage revealed a large variability between tumors (72.4 ± 6.1%). Moreover, around 78 ± 16% of vessels were positive for albumin and total albumin area took up approximately 26.5% of total tumor area (26.5 ± 4.8%), indicating the loss of structural integrity in GBM vasculature (**Figures 2H–J**).

Upon comparing the histology of GBM and MBM tumors, presumed to be at equivalent developmental stages based on growth profiles at day 14 for MBM and day 22 for GBM, we observed a marked difference in vessel density and vessel area fraction. Specifically, the vessel density in GBM was found to be approximately two times higher than that in MBM (***p = 0.0009; **Figures 2D, E**). Similarly, the mean vessel area was significantly lower in melanoma (*p = 0.01; **Figure 2G**), whilst there was no significant difference in mean vessel size (p = 0.5397; **Figure 2F**). GBM presented with significantly higher desmin-positive pericyte coverage than B16 tumors (**p = 0.001; **Figure 2H**). Simultaneously, there was significantly higher leakiness for albumin in the GBM vasculature (**p = 0.0077 and *p = 0.0149; **Figures 2I, J**).

**Figure 3:**
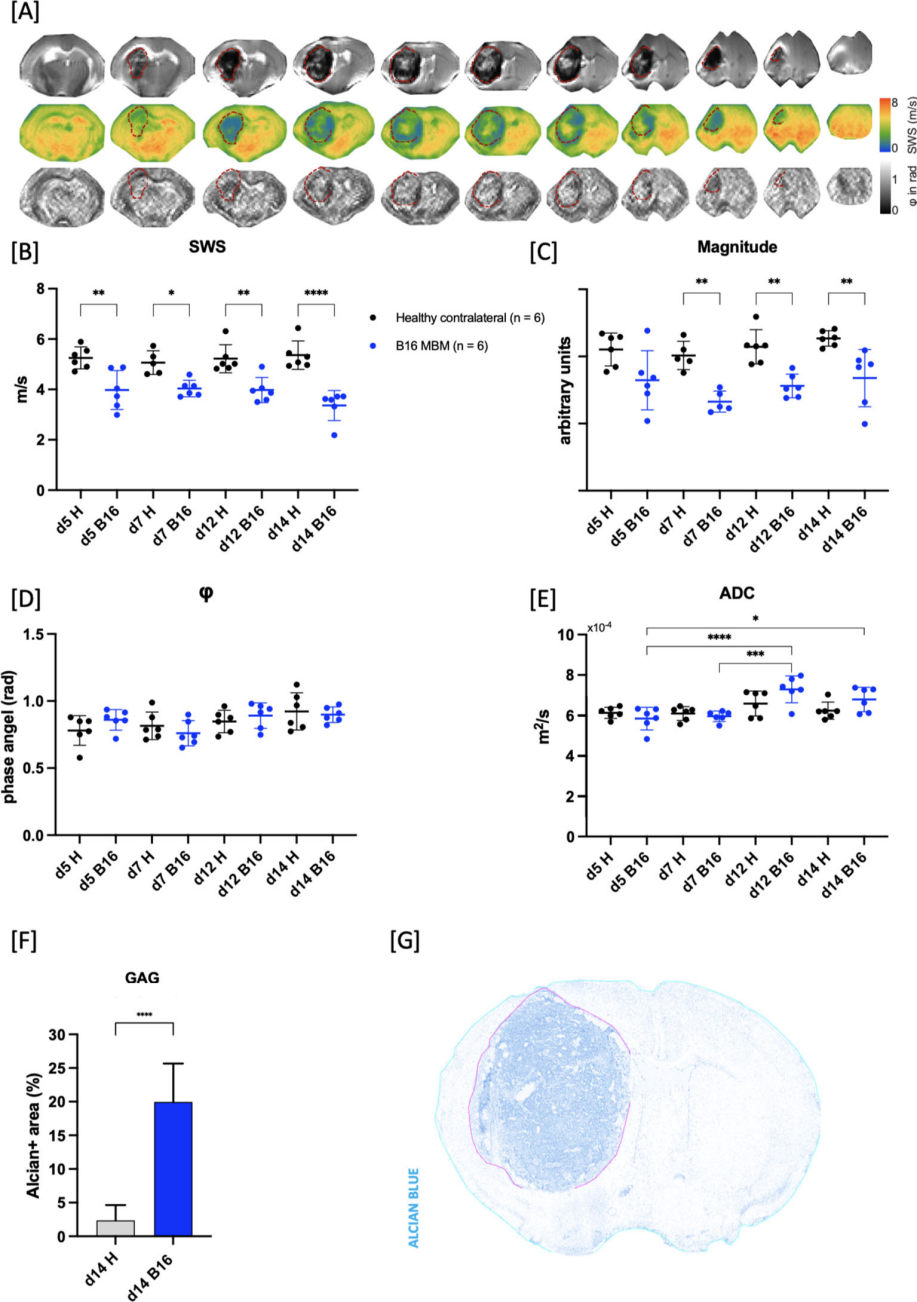
Assessment of viscoelastic properties of growing intracranial B16 melanoma. **(A)** Representative magnitude image series (upper row) with corresponding SWS maps (middle row) and φ maps (lower row) of B16 melanoma brain metastases (MBM; B16 in graphs) on day 14 demonstrating softer tissue properties of the tumor (outlined in red dotted line) compared to healthy brain tissue (adjacent). **(B)** MBM had reduced shear wave speed (SWS) from day 5 after implantation in comparison to healthy tissue of contralateral hemisphere (H in graphs; **p = 0.0023 for d5, *p = 0.033 for d7, **p = 0.0031 for d12, ****p < 0.0001 for d14). **(C)** Starting from day 7 after implantation, magnitude images displayed increasing hypointensity of tumor compared to healthy brain tissue. This resulted in relative changes of 32.5% on day 7, 28% on day 12, and 24% on day 14 (**p = 0.0042 for day 7, **p =0.0092 for day 12, **p = 0.0081 for day 14). **(D)** No significant difference in φ values when compared to healthy brain tissue or within the tumor itself were evident. **(E)** No significant difference in ADC values obtained from DWI was found between MBM and healthy brain tissue. However, ADC values within the tumor significantly rose on days 12 and 14 compared to days 5 and 7, indicative of chronic haemorrhage (****p < 0.0001 for d5 vs. d12, *p = 0.0191 for d5 vs. d14, ***p = 0.0003 for d5 vs. d14). **(F)** Alcian-Blue positive area was significantly higher in the tumor area compared to healthy brain tissue (****p < 0.0001). **(G)** Representative image of Alcian/Pas stained section, deconvoluted for Alcian blue showing tumor area (magenta-colored line) and healthy tissue (cyan-colored line).

**Figure 4:**
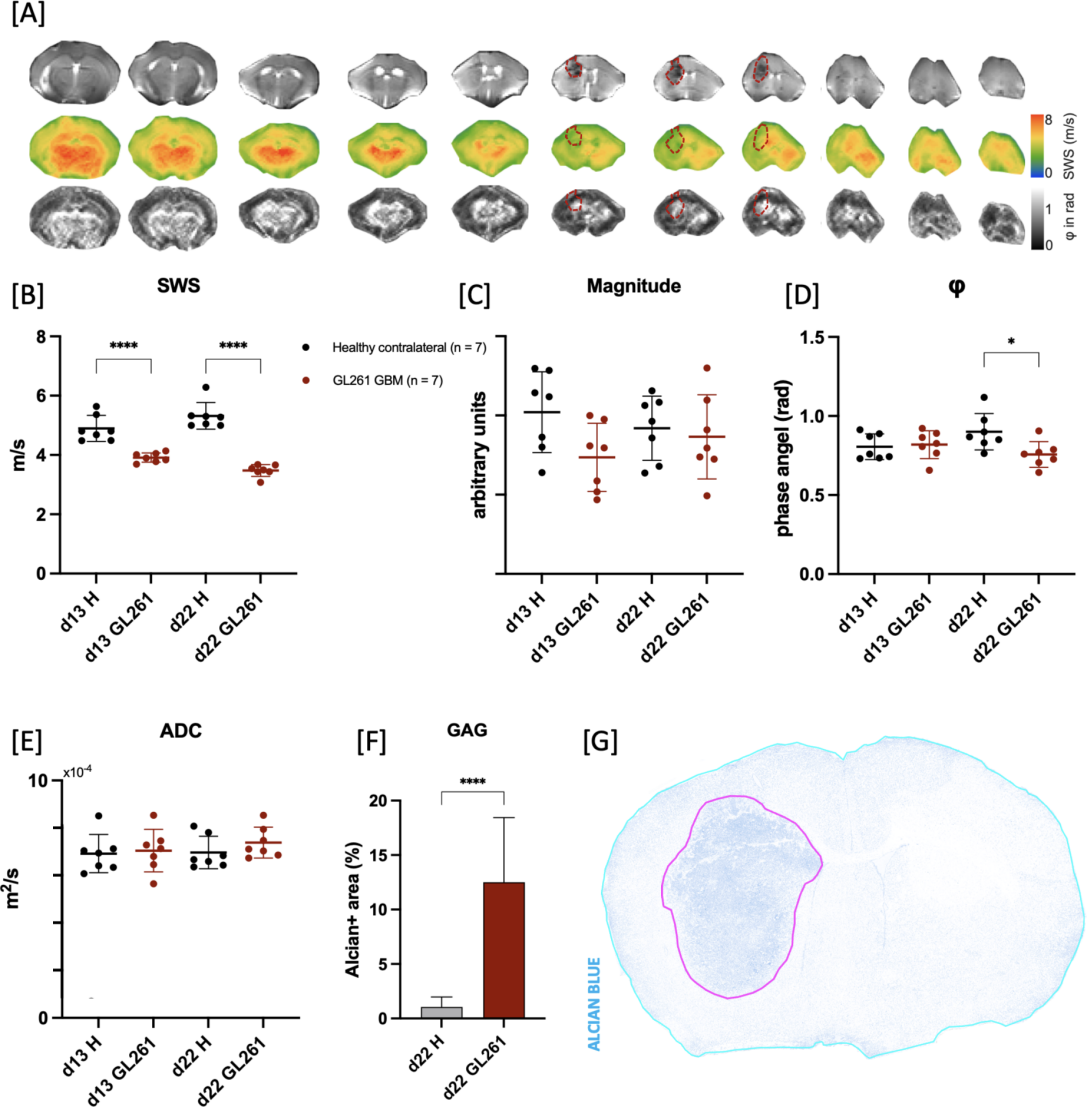
Assessment of viscoelastic properties of growing GL261 glioblastoma. **(A)** Representative magnitude image series (upper row) with corresponding SWS maps (middle row) and φ maps (lower row) of GL261 glioblastoma (GBM; GL261 in graphs) on day 22 after tumor cell implantation demonstrating softer tissue properties of the tumor (outlined in red dotted line) compared to healthy brain tissue (adjacent). **(B)** Shear wave speed (SWS) in GBM was lower compared to healthy brain tissue of contralateral hemisphere (H in graphs) on both observation timepoints indicating softer tissue properties of the tumorous tissue (****p < 0.0001 for both timepoints). **(C)** Evaluation of magnitude images showed no significant differences between the GL261 tumor and healthy brain tissue across all timepoints. **(D)** No significant alterations were found in φ values between tumor and contralateral healthy brain on day 13 after implantation. On day 22 the φ values of tumor were significantly lower than adjacent healthy brain tissue (*p = 0.0237). **(E)** ADC values derived from DWI showed no significant difference between GBM and healthy brain tissue or within the tumor tissue on both observation timepoints. **(F)** GAG-related water content in GBM was significantly higher than in healthy tissue on day 22 after implantation (****p < 0.0001). **(G)** Representative coronal section of brain on day 22 stained with Alcian/Pas deconvoluted for Alcian blue, highlighting the higher GAG content in tumor (magenta-colored line) compared to adjacent healthy brain tissue (cyan-colored line).

Furthermore, the analysis of stained sections with Alcian Blue/PAS staining revealed a significantly higher GAG-related water content in the GBM and MBM compared to healthy brain tissue as assessed by comparing fraction of GAG covered area in tumor to healthy brain tissue of contralateral brain (healthy: 2.36 ± 0.83% vs. B16: 19.9 ± 5.6% and healthy: 1.05 ± 0.91% vs. GL261: 12.5 ± 5.9%, ****p < 0.0001 for both; **Figures 3F, G** for MBM; **Figures 4F, G** for GBM).

### 3.2 Tumor biomechanical properties are distinct from healthy brain tissue

Biomechanical properties of both tumor entities were assessed. The mean difference of SWS between repeated measurements for all timepoints and tumors entities was 0.13 ± 0.04 m/s. In the B16 MBM mouse model, the tumors exhibited significantly softer tissue properties at all timepoints compared to healthy brain tissue as shown by lower SWS values (healthy: d5: 5.20 ± 0.40 m/s, d7: 5.06 ± 0.47 m/s, d12: 5.20 ± 0.56 m/s, d14: 5.36 ± 0.56 m/s vs. B16: d5: 3.97 ± 0.77 m/s, d7: 4.03 ± 0.32 m/s, d12: 3.98 ± 0.50 m/s, d14: 3.36 ± 0.60 m/s; **p = 0.0023 for d5, *p = 0.033 for d7, **p = 0.0031 for d12, ****p < 0.0001 for d14; **Figures 3A, B**). Notably, no significant changes in tumor SWS were detected as the tumor progressed (d5 vs. d7, d5 vs. d12, d7 vs. d12: p>0.99 for all; d5 vs d14: p = 0.5710; d7 vs. d14: p = 0.3720; d12 vs. d14: p = 0.5634). The magnitude images revealed a progressive increase in tumor hypointensity compared to healthy contralateral tissue, beginning on day 7 after tumor cell implantation. This resulted in relative changes of 32.5% on day 7, 28% on day 12, and 24% on day 14 (**p = 0.0042 for day 7, **p = 0.0092 for day 12, **p = 0.0081 for day 14; **Figures 3A, C**). Within the tumor, areas of varying intensity were evident, especially at day 14: central hyperintense areas were surrounded by hypointense regions, with localization coinciding with peritumoral hemorrhage. Additionally, no significant difference in φ values was observed compared to healthy brain tissue (p>0.99 for all; **Figures 3A, D**). No significant alterations of ADC values obtained from DWI were found between MBM tumor and healthy brain tissue (d5, d7: p>0.99 for both; d12: p = 0.1815; d14: p = 0.5894). However, ADC values within the tumor significantly rose on days 12 and 14 compared to days 5 and 7 (B16 d5: 5.9 ± 0.6 ×10^-4^ m^2^/s vs. B16 d12: 7.3 ± 0.7 ×10^-4^ m^2^/s, ****p < 0.0001; B16 d5: 5.9 ± 0.6 ×10^-4^ m^2^/s vs. B16 d14: 6.8 ± 0.6 ×10^-4^ m^2^/s, *p = 0.0191; B16 d7: 6.0 ± 0.3 ×10^-4^ m^2^/s vs. B16 d12: 7.3 ± 0.7 ×10^-4^ m^2^/s, ***p = 0.0003 **Figure 3E**).

The GBM cohort showed a significant decrease in tumor SWS compared to contralateral healthy brain tissue on days 13 and 22 after tumor cell implantation, indicating softer properties in the tumor (healthy d13: 4.90 ± 0.44 m/s vs. GL261 d13: 3.90 ± 0.16 m/s; healthy d22: 5.3 ± 0.5 m/s vs. GL261 d22: 3.5 ± 0.2 m/s, ****p < 0.0001 for both; **Figures 4A, B**). Simultaneously, φ was significantly decreased in tumor tissue opposed to healthy brain tissue at day 22 (healthy d22: 0.9 ± 0.1 rad/unit vs. GL261 d22 0.76 ± 0.08 rad/unit, *p = 0.0237; **Figures 4A, D**). The magnitude images indicated that the tumors were slightly more hypointense compared to the healthy brain tissue, however, analysis of relative differences revealed no significant variation between the tumor and healthy brain on any of the timepoints (**Figures 4A, C**). No substantial diffusion restriction was detected as evidenced by the ADC values (p>0.99 for all; **Figure 4E**).

In the next step, we compared the biomechanical properties of the two tumor entities to identify potential differences. Analysis of magnitude images revealed distinct patterns: GBM exhibited increased hypointensity compared to the healthy contralateral tissue, whereas MBM displayed areas of varying intensity, especially evident by day 14. However, further metrics, specifically SWS and φ values for GBM and MBM entities using segmentation masks of the whole tumor, showed no significant differences (SWS: p > 0.99 for all comparisons; φ: day 12 B16 vs. day 22 GL261: p = 0.2174; day 14 B16 vs. day 22 GL261: p = 0.4034; p > 0.99 for remaining comparisons; see **Supplementary Figure 1**). To further assess potential heterogeneity between the tumor entities, we measured the skewness of SWS (asymmetry of the SWS distribution), which revealed no significant differences (p = 0.1265 for all time points). Additionally, homogeneity based on the 2D gray-level co-occurrence matrix (GLCM, which examines the spatial relationship of pixel intensities to assess texture) showed no significant differences between both tumors (p = 0.3497). For better illustration, we included an overview of the histograms for individual tumors on day 14 for MBM and day 22 for GBM in **Supplementary Figure 2**.

## 4 Discussion

We successfully applied MRE with tomoelastography post processing, an advanced multifrequency MRI-based non-invasive imaging technique, to a preclinical intracranial B16 melanoma mouse model unveiling the initial insights of the tumor biomechanics. The findings were further compared to the well-established GL261 glioma mouse model. Our results contribute to the understanding of brain tumor biomechanics and highlight the potential of MRE for noninvasive tumor characterization, given its adaptability across length scales from mouse models to humans (13, 38). Integration of MRE with other imaging sequences in a concise protocol, was possible due to an optimized acquisition time for a full multifrequency scan of only 12 minutes. This complies with the 3R principle of “refinement”, a cornerstone of animal-centered research. The reduced acquisition time ensures seamless imaging and data collection, minimizing animal stress, and strengthening the results’ validity. Our approach offers a pivotal platform for dissecting tumor growth dynamics and biomechanics, priming advancements in targeted therapy monitoring and refined treatment planning.

### 4.1 Histological appraisal discerned the typical features of both tumor entities

Malignant intracranial tumors are associated with softer tissue properties compared to healthy brain, as corroborated by numerous studies in both human and animal models (25). The murine B16 melanoma model, which represents intracranial metastases, was chosen because of the high incidence of melanoma as a source of brain metastases (39). However, intracranial growth dynamics and biomechanical properties of the B16 cell line are less explored. Previous research using bioluminescence measurements (37) or survival analysis (40, 41) to characterize tumor growth dynamics cannot directly be compared with our model due to the divergent measurement techniques. Nevertheless, our findings on tumor growth patterns, assessed through MRI volumetric analyses, aligned with existing literature. Notably, tumors exhibited significant growth starting from day 7 after implantation. Histological characterization of intracranial melanoma tumors remains limited, but clinical studies have recognized melanoma brain metastases as the most prevalent intracranial malignancy causing intratumoral hemorrhage (30–33). In a cohort of 357 patients undergoing neurosurgical metastasis resection, Hamed et al. reported 38 (10.6%) with melanoma brain metastases, of which 73% exhibited heamorrhage signs on preoperative MRI (42). Consistent with this, our study identified hemorrhages by MRI and histology in five of six animals. Insufficient blood brain barrier (BBB) integrity, as evidenced by low pericyte coverage and high vessel leakiness, may contribute to the tendency for hemorrhage of this model.

Furthermore, the well-established GL261 glioma mouse model was employed and validated for its representativeness relative to those in the literature (43). The GL261 cells have demonstrated the ability to replicate the majority of GBM hallmarks, exhibiting rapid and aggressive growth patterns with limited invasive potential in *in vivo* settings (43, 44). Our findings substantiate the characteristic growth behavior, with a ten-fold increase in tumor volume observed between days 13 and 22 after implantation. The results pertaining to vascular supply and vessel morphology were consistent with prior findings, as shown by a mean vessel area of 12.8 ± 2.2% within the tumor area, aligning with the published range (30). Consequently, the glioma model employed in this study was representative of already published tumor models and was suitable for assessing its biomechanical properties using our MRE protocol.

Comparison of the histologic features of both tumor entities revealed faster growth dynamics of melanoma than GBM with peak volume 14 days after implantation versus 22 days in GBM. Upon histological appraisal, both entities presented tumor specific features, namely GBM with pronounced vascularization and extensive hemorrhages in melanoma. Despite comparable individual vessel size, GBM displayed higher vessel density and desmin coverage. Interestingly, although GBM presented with higher pericyte coverage, the amount of albumin positive vessels was higher, indicating higher vessel leakage in GBM than melanoma. The most accurate reason for this observation might be the extensive hemorrhage of melanoma that might falsify the results by washing out the albumin. As this paper presents initial observations of the whole tumor milieu, further analyses are required focused on detailed assessment of the different tumor components and correlation with parameters like biomechanical properties.

### 4.2 Comparison of the presented MRE technique to already published protocols: optimization of acquisition time crucial for incorporation of MRE into experimental setup

It is known that processes such as demyelination and extracellular matrix degradation result in changes of brain tissue biomechanical properties (45). While MRE can be readily incorporated into clinical routines for patients, its implementation in preclinical models presents a greater challenge due to the lack of time efficient MRE sequences. We summarized the reported elastography methods in intracranial tumor bearing animal models in **Table 2** (GBM) and **Table 3** (melanoma), highlighting MRE acquisition time range from 23 to 51 minutes per animal, which poses challenges when combined with other imaging sequences (17, 21, 22, 24). Schregel et al. reported the shortest acquisition time of 23 minutes using a G30 glioma mouse model; however, when combined with a T2-weighted MR sequence, the protocol extended to 35 minutes per animal (21). In contrast, our whole MRI protocol required a total of 25 minutes, with 12 minutes for MRE.

**Table 2:** Summary of literature on magnetic resonance elastography (MRE) for intracranial glioblastoma (GBM).

Article	Year	Tumor model	Imaging					Histological appraisal
			Scanner	MRI protocol	MRE technique	MRE acquisition time (min)	Total acquisition time (min)	
**Schregel, K et al.** (46)	2020	G9pCDH-GBM (i.c.)	BioSpec 7T animal MRI (Bruker)	1. T1 + CA (Magnevist) 2. T2 3. MRE	-TR/TE: 900/29 ms -FOV:19.2 mm -Matrix: 64x64 -9 slices -8 wave phases -Freq.: 1kHz	23	38:36	DAPI, endogenous GFP, Flouro-myelin stain, anti-murine-CD31 stain
**Li, J et al.** (24)	2019	U-87 MG GBM (i.c./s.c.) Rat luc-RG2 glioma (i.c.) D-212 MG GBM (i.c.)	7T horizontal bore MicroImaging system	1. MRE 2. T2	-TR/TE: 1001/27 ms -10x300µm -Freq.: 1 KHz	n.a.	51	H&E, picrosirius red, anti-murine-CD31 stain
**Schregel, K et al.** (21)	2018	G30 GBM (i.c.)	7T horizontal bore Bruker small animal scanner	1. T2 2. MRE	-TR/TE: 900/29 ms -FOV: 19.2 mm -Matrix: 64x64 -8 wave phases -9 slices -Freq.: 1kHz	23	35	H&E, Hoechst, Myelin, actin cytoskeleton, tubulin skeleton, anti-murine-CD31, and endogenous mCherry red protein
**Feng, Y et al.** (22)	2016	DBT GBM (i.c.)	4.7T small animal MR imaging system	1. T2 2. MRE	-FOV: 16mm -4 wave phases -29 slices -Freq.: 1.8 kHz	n.a.	23–160	none
**Jamin, Y et al.** (17)	2015	U-87 MG GBM RG2 glioma (i.c.)	7T Bruker horizontal bore Micro Imaging system	1. MRE 2. T2	-TR/TE: 1001/38 ms -FOV:1.92x1.92 cm^2^ -10 slices -8 wave phases -Freq.: 1 kHz	51	n.a.	H&E, picrosirius red, anti-murine-CD31, Luxol fast blue staining

CA, Contrast Agent; CD31, Cluster of differentiation for endothelial cells; DAPI, 4′,6-diamidino-2-phenylindole (nuclear DNA); FOV, Field of View; Freq, Frequency; GBM, Glioblastoma multiforme; GFP, green-fluorescent protein; H&E, Hematoxylin and eosin staining; i.c, intracranial; s.c., subcutaneous; MRE, Magnetic Resonance Elastography; MRI, Magnetic Resonance Imaging.

**Table 3:** Summary of literature on elastography in melanoma model.

Article	Year	Tumor model	Imaging					Histological appraisal
			Scanner	elastography protocol	elastography technique	elastography acquisition time (min)	Total acquisition time (min)	
**Riegler, J et al.** (47)	2018	B16F10 melanoma (s.c.)	Acuson S2000 ultrasound system	1. ARFI examination of tumor 2. CEUS imaging (SIMB4-5 as CA)	-FOV: 3x2 square cm (ARFI) and 3,5x3 cm2 (CEUS) -Freq.: 14 MHz and 8 MHz -Power: 27% -in plane resolution: 50 µm (ARFI) and 90 µm (CEUS) -300 µm slice thickness	15	15	Endomucin, Lectin, DAPI, TUNEL, F4-80, CD3, HABP, PDGFR-α, SMA, DAPI, Collagen I, III, IV

ARFI, Acoustic Radiation Force Imaging; CA, Contrast Agent; CEUS, Contrast Enhanced Ultrasound; CD3, Cluster of differentiation for T-cells; DAPI, 4′,6-diamidino-2-phenylindole (nuclear DNA); F4/80, marker for Macrophages; FOV, Field of View; Freq., Frequency; HABP, Hyaloronan binding protein; H&E, Hematoxylin and eosin staining; PDGFR-α, Platelet derived Growth Factor alpha; s.c., subcutaneous; SMA, smooth muscle actin; TUNEL, Terminal deoxynucleotidyl transferase dUTP nick end labeling (marker for Apoptosis).

### 4.3 Malignant tumors in the brain present distinct biomechanical properties as opposed to healthy brain tissue

In GBM and MBM, we analyzed tumor biomechanical properties at two and four experimental time points, respectively. We observed softer tissue properties in GBM, consistent with findings in higher tumor grades and corroborating previous patient (16) and murine studies (17). Schregel et al.’s longitudinal G30 glioma study using MRE yielded similar results (21). Intriguingly, the authors reported biomechanical inhomogeneities within GBM: areas of high vascularity and cell density were stiffer, contrasting softer necrotic zones. While visual inspection of the derived SWS maps hinted at heterogeneity in tumor subregions, a quantitative assessment employing standard deviation, skewness and GLCM analysis as indices of SWS variability did not detect discernible differences. This is possibly due to the whole tumor masks used in this investigation. This approach could have hidden crucial details or weakened the impact of our findings. Similarly, qualitative analysis of T2/T2*w contrast as surrogate for water content in the tissue, did not reveal any significant changes either. Subsequent studies will focus on localized analyses to discern and detail these intratumoral variations.

Another aspect is the tissue viscosity as quantified by the loss angle φ. The observed decrease in tumor φ at day 22 after implantation, together with high GAG content highlights the anomalous soft-solid property of this tumor entity. This is in line with findings in human GBM samples by Streitberger et al. where it was shown that GBMs become softer and more solid in favor of infiltrative growth (35).

In contrast, our examination revealed that the apparent diffusion coefficient (ADC) values, when compared against healthy brain tissue, didn’t show any significant deviations. This is consistent with a patient study by Hakyemez et al. (48). In their analysis of 48 patients with histologically confirmed GBM, the authors were able to differentiate the commonly observed typical presentations (without diffusion restriction) and the less frequent atypical ones (displaying diffusion restriction). Only 12% of the total fell into the atypical category allowing the conclusion that DWI alone is not helpful and needs to be corroborated by further imaging investigations.

In the MBM model, lower SWS values were observed throughout tumor development, indicative of softer tumor tissue properties, aligning with the histological observation of extensive hemorrhages, which is a characteristic feature of melanoma brain metastases. The reduced tissue stiffness can be further attributed to the compromised BBB integrity, as evidenced by low pericyte coverage and high vessel leakiness observed histologically. Contrast enhancement patterns in MRI investigations underpined this interpretation: while GBM homogeneously enhanced, contrast enhancement in B16 MBM was patchy within the tumor with an enhancing rim around the tumor, representing extensive hemorrhages and pronounced BBB disruption. These findings were corroborated by the analysis of the magnitude images, which showed increasing tumor hypointensity starting from day 7 after implantation compared to healthy contralateral tissue. By day 14, tumors displayed central hyperintense areas surrounded by hypointense regions, coinciding with peritumoral hemorrhage. Comparing SWS and T2/T2*w signal intensity revealed that hypointense areas in magnitude images corresponded to softer tissue properties, suggesting these areas represented hemorrhage, while hyperintense areas represented solid tumor. Cildag et al. assessed the relationship between post-procedural hemorrhage and alterations in shear wave speed in patients undergoing percutaneous renal parenchyma biopsy. Similar to our results, the authors found significantly lower mean shear wave velocity in patients with post-procedure hemorrhage compared to those without hemorrhage (49). By days 12 and 14, increased ADC values were evident through DWI, aligning with histological findings of chronic hemorrhage and varying vessel coverage. Riegler et al. is currently the only study examining melanoma biomechanical properties using elastography, specifically focusing on subcutaneously implanted B16 melanoma cells (47). Their classification of melanoma as a soft tumor aligned with our findings. Notably, they also observed that the stiffness in the rapidly proliferating B16F10 melanomas remained consistent. Further analysis showed negative correlations between myofibroblast or vessel density and stiffness in solid tumors and positive correlations in soft tumors like B16F10, potentially due to differences in perfusion (47). With regard to brain metastases originating from other tumor entities, two studies provided initial insights into the biomechanical properties of breast cancer metastases (17, 24). Li et al. found that intracranial metastases from MDA-MB-231 breast cancer cells were softer than subcutaneous tumors of the same cell line (24). Similarly, Jamin et al., while analyzing the same breast cancer model, observed tumors with reduced viscosity and elasticity relative to the surrounding brain tissue (17). In line with these results, our data presented intracranial MBMs as notably softer than healthy brain tissue, underscored by increased hypointensity of tumor compared to the healthy tissue in magnitude images, suggestive of pronounced hemorrhage. However, the sparse investigations on BM models underscore the urgent need for research in this field.

Although both tumor entities presented with similar biomechanical properties, combining the results with histological and imaging data allows for further conclusions regarding both tumors. Both BMB and GBM were found to be softer than healthy brain tissue, with no significant differences in SWS values. This finding aligned with the work of Reiss-Zimmermann et al., who investigated the biomechanical properties of various brain pathologies in a clinical context, concluding that the different entities were not distinguishable based on their mean SWS values (18). Considering the histological and imaging features of both tumors, the softness of GBM was likely due to pronounced vascularization and GAG content, whereas the softness of melanoma was due to pronounced hemorrhages, evident in histological and MRI assessments. Additionally, the GAG-covered area was found to be higher in both tumors compared to the contralateral healthy brain tissue, further corroborating the soft tissue properties of the tumors. These findings underscore the complementary nature of MRE with other widely established techniques. By combining MRE with traditional histological methods, we aim to gain a more nuanced understanding of tumor characteristics. This integrated approach is crucial for developing more targeted and effective treatment strategies and may serve as guidance for future therapeutic approaches, including considerations like radiation planning (50).

### 4.4 Limitations

The findings presented here, despite their encouraging nature, have certain limitations. Primarily, the analyses were conducted using whole tumor masks which offered a comprehensive overview of the tumor’s biomechanical properties. However, this approach somewhat masked the heterogeneity within the tumor, potentially accounting for the lack of significant alterations of the tumor parameters or the absence of clear distinctions between the two tumor types. As we aim to offer a broader characterization of the tumor types, we plan to proceed with more in-depth subregional analyses and to correlate these to the irradiation treatment response in a further study. While using a fast MRE technique to improve acquisition speed and efficiency has shown to be very powerful, this approach also has inherent limitations. Subject motion during the measurement process can result in blurring and complex image artifacts, particularly for segmented sequences. Additionally, the use of echo-planar imaging (EPI) in MRE, though beneficial for its speed, introduces distortions due to susceptibility-induced B0 inhomogeneities. These distortions are especially pronounced at higher image resolutions and field strengths. Therefore, correcting for EPI distortions is crucial as it enhances anatomical localization and significantly increases the statistical power of multisubject studies. Another limitation is the growth pattern of the GL261 GBM, which demonstrates as a bulk mass and does not exhibit the same infiltrative characteristics as human GBM, thus hindering the translation to the human condition. Nevertheless, the lack of infiltrative growth represents an advantage in regard to the delineation of the tumor without capturing the healthy brain tissue, which would further distort the results. In addition, histological correlation was feasible only for a single time point. This posed a challenge for the in-depth evaluation of the longitudinal changes in tumor biomechanical properties. A contributing factor to this difficulty was the overall limited number of animals in the experiment, which precluded sacrificing some for histological correlations. However, adhering to the 3R principle, we strategically adopted a sequential study setup. This maximized data yield from each animal, reducing the overall animal usage while maintaining the integrity and depth of our investigations.

### 4.5 Conclusion

In this study, we systematically analyzed the *in vivo* viscoelastic properties of MBM and compared them with those of the established GBM mouse model. Our primary goal was to introduce MRE with tomoelastography post processing as a method for brain tumor characterization. Our findings revealed that, unlike solid tumors in other organs, aggressive brain tumors exhibit softer mechanical properties even in their early stages and display distinct characteristics. Both MBM and GBM showed overall softer tissue properties compared to healthy brain tissue, with imaging and histological analyses indicating different microstructural reasons for this mechanical tumor signature. In MBM, chronic hemorrhages were observed, corroborated by increased ADC values in DWI and the appearance on T2/T2*w imaging contrast, while the softness of GBM was likely due to pronounced vascularization and GAG content. These results highlight the complementary nature of MRE and its potential to enhance our understanding of tumor characteristics when used alongside established techniques. The biomechanical assessment of an *in vivo* intracranial B16 melanoma mouse model and its comparison to the GL261 glioma model, further enhances the validity of the results. This research underscores the necessity of using diverse preclinical models for a thorough analysis of tumors and stresses the importance of further studies to identify factors influencing changes in tumor biomechanical properties. Multiparametric MRI, which includes quantification of tissue stiffness, fluidity, water diffusion, and relaxation time-dependent imaging contrasts, combined with established techniques like conventional MRI and histopathological appraisal, offers significant promise for improving brain tumor diagnosis, prognosis, and treatment strategies. This comprehensive approach could lead to better clinical outcomes and a deeper understanding of brain tumor pathophysiology.

## Data Availability

The original contributions presented in the study are included in the article/**Supplementary Material**. Further inquiries can be directed to the corresponding author/s.
