# Supplementary figures and images for: In vivo characterization of brain tumor biomechanics: magnetic resonance elastography in intracranial B16 melanoma and GL261 glioma mouse models
Source: Front Oncol. 2024 Sep 11;14:1402578. doi: 10.3389/fonc.2024.1402578 (PMC11422132; doi:10.3389/fonc.2024.1402578)

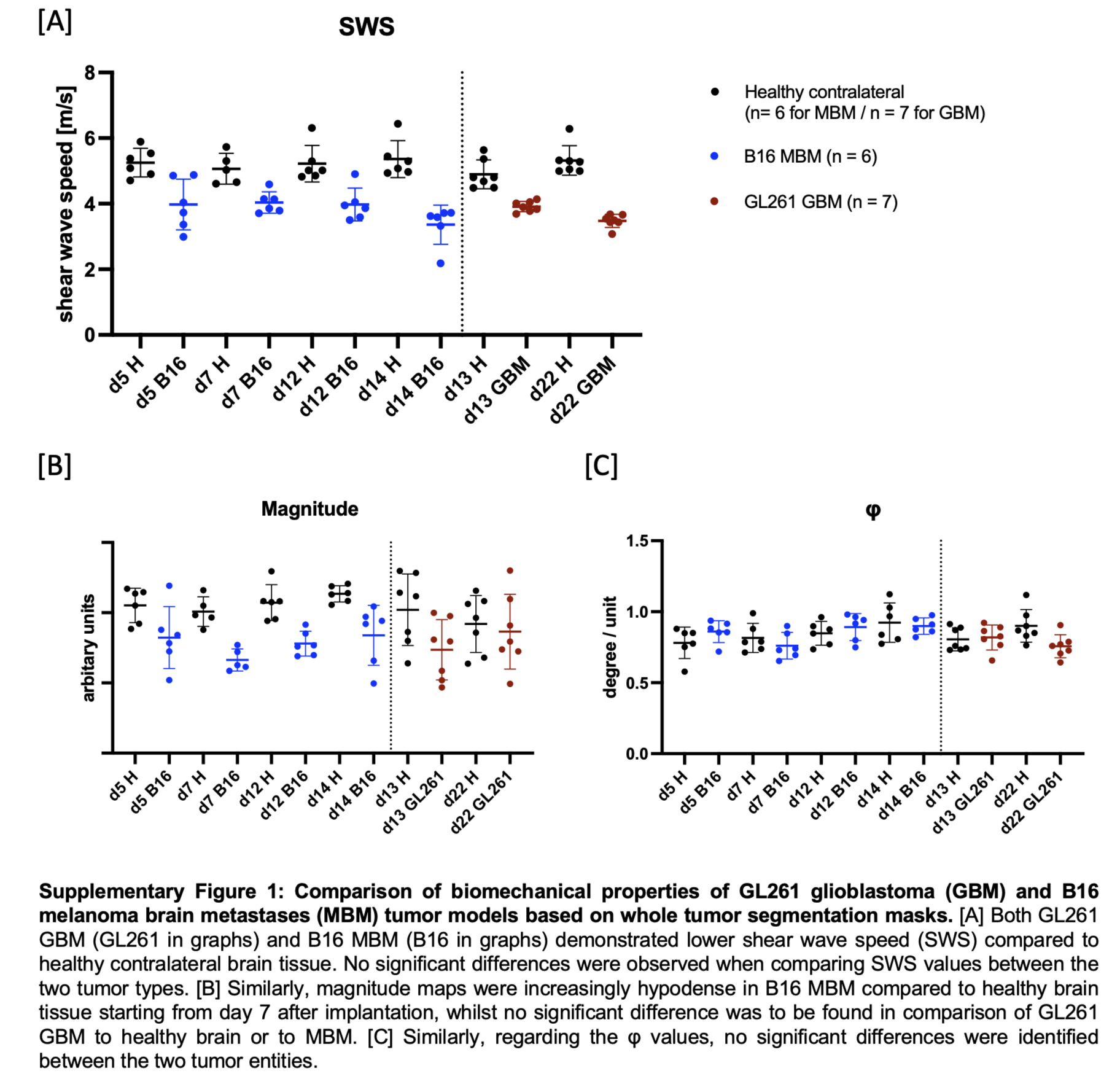

Supplement: Supplementary file 1 [file Image1.tiff]

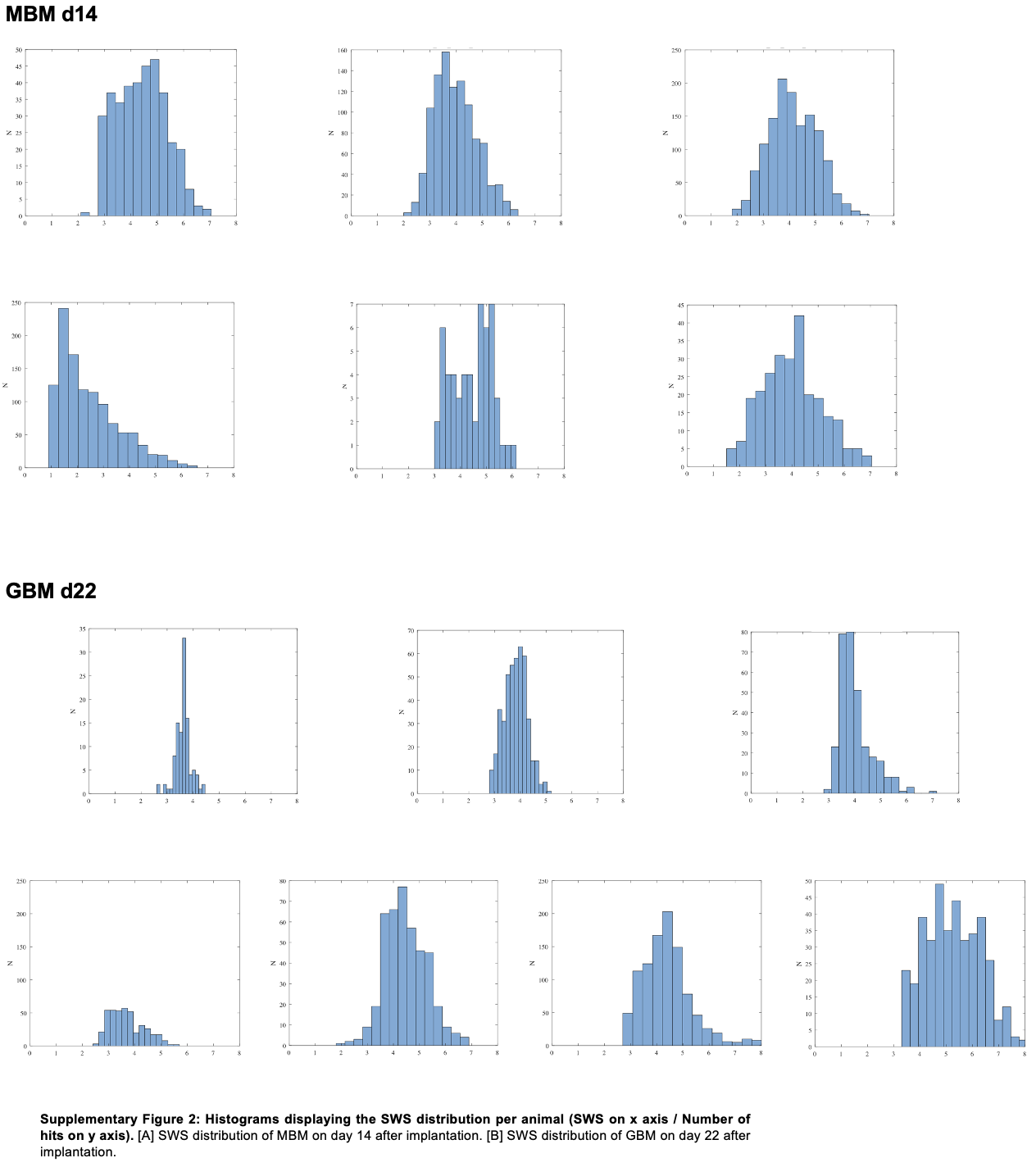

Supplement: Supplementary file 2 [file Image2.tiff]
